# Autophagy under attack

**DOI:** 10.7554/eLife.14447

**Published:** 2016-02-23

**Authors:** Paul de Figueiredo, Marty Dickman

**Affiliations:** 1 Norman Borlaug Institute, Department of Veterinary Pathobiology and Department of Microbial Pathogenesis and Immunology, Texas A&M University, College Station, United States; 2 Norman Borlaug Institute and the Department of Plant Pathology and Microbiology, Texas A&M University, College Station, United Statesmbdickman@tamu.edu

**Keywords:** nicotiana benthamiana, phytophthora infestans, autophagy, effectors, irish potato famine, late blight disease, Other

## Abstract

Pathogens target proteins involved in autophagy to inhibit immune responses in plants.

**Related research article** Dagdas YF, Belhaj K, Maqbool A, Chaparro-Garcia A, Pandey P, Petre B, Tabassum N, Cruz-Mireles N, Hughes RK, Sklenar J, Win J, Menke F, Findlay K, Banfield MJ, Kamoun S, Bozkurt TO. 2016. An effector of the Irish potato famine pathogen antagonizes a host autophagy cargo receptor. *eLife* **5**:e10856. doi: 10.7554/eLife.10856

**Image** PexRD54 is a protein that interferes with the process that plant cells use to destroy damaged or unwanted proteins

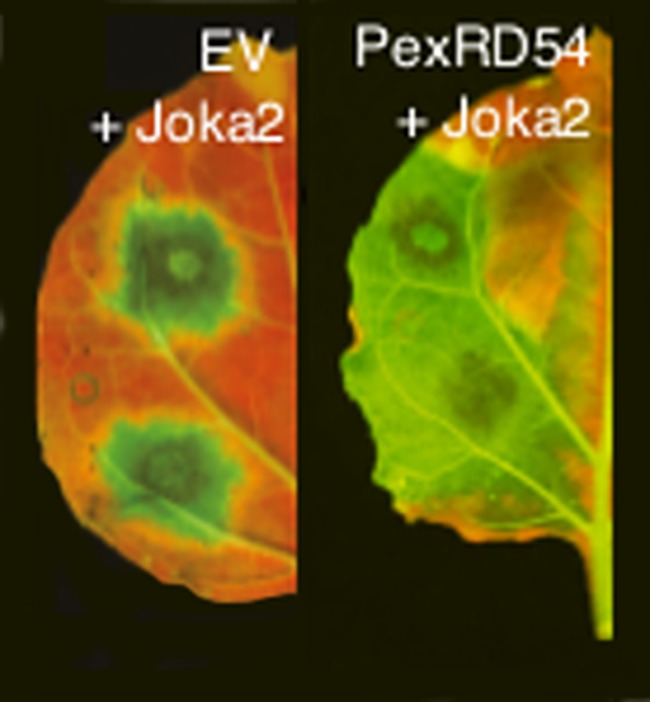

The Irish potato famine was responsible for more than one million deaths and the emigration of one million people from Europe in the 1840s (Andrivon, 1996). Today, the microbe that caused the famine, an oomycete called *Phytophthora infestans*, continues to cause serious outbreaks of disease in potato crops. Traditional control measures, such as fungicides and breeding for resistance, often have only marginal success in combating the disease, especially when the climate favors the growth and development of *P. infestans* (Fry and Goodwin, 1997). Now, in eLife, Sophien Kamoun, Tolga Bozkurt and colleagues – including Yasin Dagdas and Khaoula Belhaj as joint first authors – reveal how one of the proteins produced by *P. infestans* manipulates host plant cells to weaken their defenses (Dagdas et al., 2016).

It is well established that plant pathogens secrete proteins and small molecules – collectively known as effectors – that can interfere with plant defenses and make it easier for pathogens to infect and spread (Djamei et al., 2011; de Wit et al., 2009; Rovenich et al., 2014; Gawehns et al., 2014). However, as part of an ongoing arms race between plants and pathogens, some effectors are recognized by proteins in the host plant, which triggers immune responses that act to contain the infection. Relatively little is known about how effectors interfere with plant defenses. In particular, the identities of the plant molecules that are targeted by the effectors, and details of how the effectors are transported into plant cells, remain unclear.

The success of *P. infestans* as a pathogen is largely due to its ability to secrete hundreds of different effectors. Now, Dagdas, Belhaj et al. – who are based at the Sainsbury Laboratory, the John Innes Centre and Imperial College – report how they carried out a screen for plant molecules that interact with effectors from *P. infestans *(Dagdas et al., 2016). The experiments were carried out in the leaves of tobacco, which is a commonly used plant model, and show that an effector called PexRD54 targets a process called autophagy in plant cells.

Autophagy is a complex “self-eating” process that occurs when plant and other eukaryotic cells experience certain stresses – for example, due to a shortage of nutrients or a change in environmental conditions. During autophagy, cell material is broken down to supply the building blocks needed to maintain essential processes (Li and Vierstra, 2009). More recently, autophagy has been implicated in a variety of other situations, including restricting the growth and spread of invading microbes. A growing body of evidence suggests that autophagy plays a dual role both in promoting the survival of cells and in triggering cell death.

During autophagy, cell materials are sequestered by structures called autophagosomes and then delivered to acidic cell compartments where the material is degraded and recycled. In addition to supporting the bulk degradation of cell materials, it was recently shown that autophagy allows the selective removal of cellular components that are damaged or no longer needed. In selective autophagy, the sequestered material is loaded into autophagosomes by specific interactions between receptor proteins and specific autophagy proteins, such as the ATG8 proteins (Stolz et al., 2014, Lamb et al., 2013).

Dagdas, Belhaj et al. found that PexRD54 interferes with the activity of a potato cargo receptor called Joka2. PexRD54 out-competes Joka2 to bind to an ATG8 protein and stimulate the formation of an autophagosome in the plant cell (Figure 1). In doing so, the oomycete cleverly reduces the loading of specific types of cargo into autophagosomes and thus limits the plant defense response.

**Figure 1.**
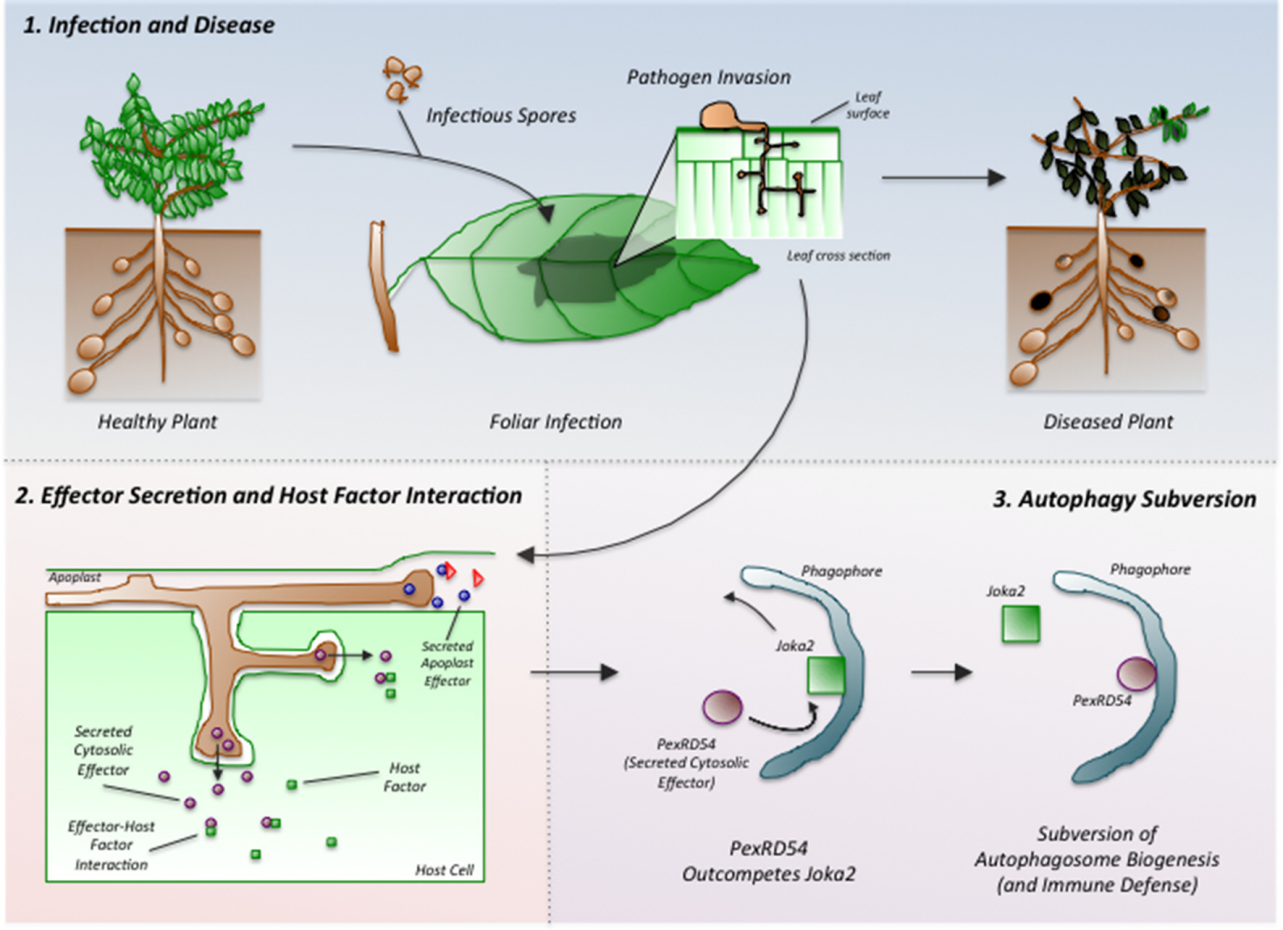
*Phytophthora infestans* interferes with the immune responses of potato plants. Spores of *P. infestans* land on the leaves of potato plants and germinate (top middle). The growing fungus enters the leaves and spreads around the plant, leading to disease (top right). Proteins called effectors are released from the pathogen and some are taken into the cells of the host plant (bottom left). These effectors (purple ovals) interact with host factors (green squares) to promote the progression of the disease. Dagdas, Belhaj et al. found that a *P. infestans* effector called PexRD54 (purple oval; bottom right) out-competes a plant cargo receptor known as Joka2 (green square) on the surface of a membrane structure called a phagophore, which eventually becomes an autophagosome. In this way, PexRD54 prevents the loading of cargo proteins into autophagosomes and inhibits plant defenses.

The reported observations expand upon studies of mammalian pathogens that also harbor effectors that interfere with autophagy (Table 1). Taken together, this work provides a template for future investigations into the ways in which effectors subvert host plant defenses. However, a number of interesting questions remain unanswered. For example, how do cargo receptors work? How are they regulated? What is the nature of the cargo in the autophagosomes and how does it regulate immune responses? In addition, our understanding of the mechanisms that control selective autophagy remain incomplete. How is the selectivity regulated, and what other cell mechanisms might be subverted by effectors? *Phytophthora* diseases can have devastating effects, but as this study illustrates, they can also illuminate and advance our understanding of fundamental cellular processes.

**Table 1.** Mammalian pathogens that express proteins that interfere with host autophagosome biogenesis or function.

Domain	Pathogen	Host	Effector	Activity	Refs
Virus	HIV virus	human	Nef1	Inhibits host autophagy	Campbell et al., 2015
	CMV virus	human	Trs1	Inhibits host autophagy	Chaumorcel et al., 2012
	Dengue virus	mammal	NS4A	Upregulation of autophagy	McLean et al., 2011
Bacteria	*Legionella*	mammal	RavZ	Cleaves an Atg8 protein from pre-autophagosomes	Choy et al., 2012; Horenkamp et al., 2015
	*Coxiella*	mammal	Cig2	Disrupts interactions between acidic compartments and host autophagosomes	Newton et al., 2014
	*Salmonella*	mammal	SseL	Inhibits selective autophagy of cytosolic aggregates	Mesquita et al., 2012
	*Anaplasma phagocytophilum*	mammal	Ats-1	Hijacks a pathway that activates autophagy to promote its growth inside cells	Niu et al., 2012
	*Vibrio parahemolyticus*	mammal	VopQ	Creates pores in acidic compartments in host cells	Sreelatha et al., 2013
Eukaryote	*Phytophthora*	plant	PexRD54	Inappropriately activates the formation of autophagosomes	Dagdas et al., 2016
